# T1 mapping is abnormal before decline in EF in patients with Becker and Duchenne muscular dystrophy

**DOI:** 10.1186/1532-429X-15-S1-P149

**Published:** 2013-01-30

**Authors:** Jonathan H Soslow, Bruce M Damon, William B Burnette, David Parra, Larry W Markham

**Affiliations:** 1 Pediatrics, Division of Pediatric Cardiology, Vanderbilt University Medical Center, Nashville, TN, USA; 2 Neurology, Vanderbilt University Medical Center, Nashville, TN, USA; 3 Radiolology and Radiological Sciences, Molecular Physiology and Biophysics, and Biomedical Engineering, Vanderbilt University Medical Center, Nashville, TN, USA; 4 Internal Medicine and Pediatrics, Division of Cardiology, Vanderbilt University Medical Center, Nashville, TN, USA

## Background

Patients with Duchenne muscular dystrophy (DMD) develop cardiomyopathy (CM) at an earlier age compared to Becker muscular dystrophy (BMD), but the age of onset within each diagnosis is variable. A method to predict CM onset associated with dystrophinopathy could alter therapeutic approaches and improve outcomes. Shortened post-contrast T1 relaxation times are an early marker of myocardial fibrosis and are abnormal in patients with DMD as described in our previous work. To our knowledge, T1 mapping has not been evaluated in BMD patients. We hypothesized that patients with BMD would also have abnormal T1 times and that these abnormalities would precede LV dysfunction.

## Methods

Twenty-six CMR scans from these 2 dystrophinopathy populations (N=5 BMD and N=21 DMD) were compared with 10 CMR scans from control patients without cardiovascular disease. T1 maps were created from the Look-Locker sequence, obtained 10 minutes after gadolinium injection, using MRMap. Using MatLab, T1 times were obtained for every voxel in 6 standard myocardial segments in the short axis at the level of the papillary muscles. Mean T1 times were compared between BMD, DMD and controls using a Kruskal Wallis test. A subset of patients with normal LVEF and dystrophinopathy (BMD or DMD) was compared to controls using a Mann-Whitney U test.

## Results

**Figure 1:**
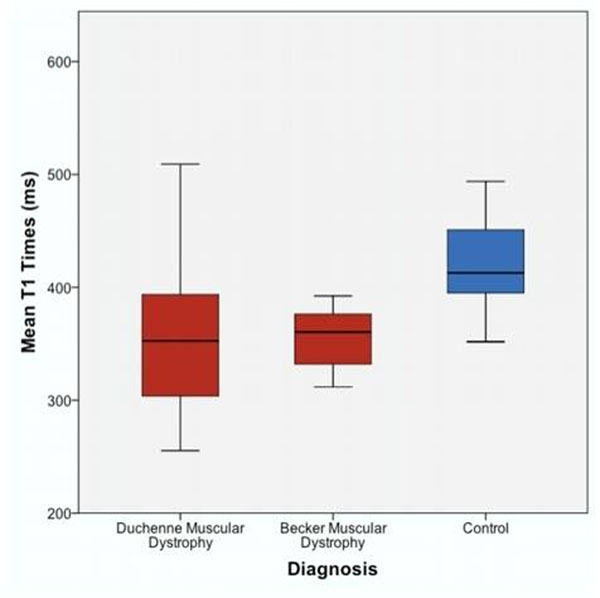
Mean T1 times by diagnosis. Patients with Duchenne muscular dystrophy (DMD) and Becker muscular dystrophy (BMD) have significantly shorter mean post-contrast T1 relaxation times compared with controls.

**Figure 2:**
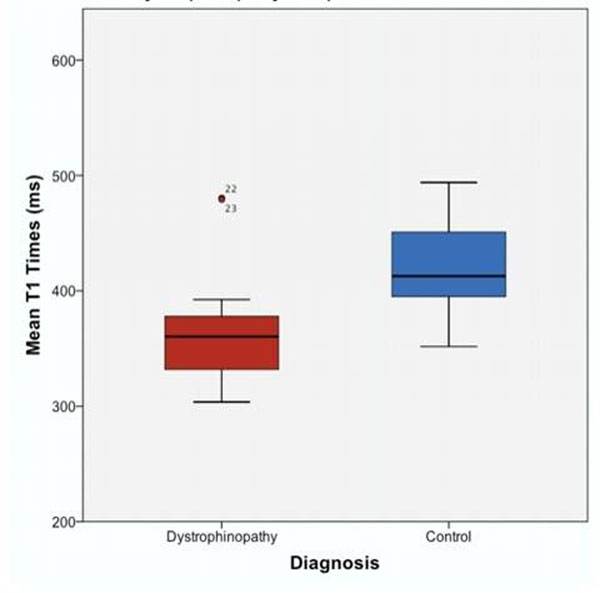
Mean T1 times in patients with normal LVEF and dystrophinopathy compared to controls. A subset of patients with Becker muscular dystrophy (BMD) and Duchenne muscular dystrophy (DMD) and normal left ventricular ejection fraction (LVEF) also had a significant decrease in post-contrast T1 when compared to controls. This suggests that myocardial T1 mapping can detect myocardial damage in patients with dystrophinopathy prior to a decline in LVEF.

The mean age of patients was 25.4 ± 8.1 years in the BMD group, 15.7 ± 4.3 years in the DMD group, and 16.9 ± 1.3 years in controls. One patient with BMD and 13 patients with DMD had LVEF < 55%. Two patients from both BMD and DMD had LV dilatation. BMD and DMD patients had significantly shorter mean post-contrast T1 compared with controls (BMD: 355 ms, 95% CI (314, 395), DMD:357 ms, 95% CI (323, 390), control: 420 ms, 95% CI (389, 451), p=0.035) (Figure 1). The subset of BMD and DMD patients and normal LVEF also had a significant decrease in post-contrast T1 when compared to controls (-52.4 ms, 95% CI (-7.2, -97.5), p=0.018) (Figure 2).

## Conclusions

Myocardial post-contrast T1 relaxation times in BMD and DMD are significantly shorter than in controls. They remain significantly decreased in the subset of patients with normal LVEF, suggesting that CMR may have prognostic utility in detecting subclinical myocardial damage associated with loss of dystrophin prior to a decline in LVEF. Further longitudinal study is necessary to evaluate whether T1 times can predict future onset of CM in BMD and DMD.

## Funding

none

